# 458. Risk of COVID-19 and vaccine immunogenicity in pediatric solid organ transplant recipients – a single center study in South Korea

**DOI:** 10.1093/ofid/ofad500.528

**Published:** 2023-11-27

**Authors:** Jee Yeon Baek, Kyoung Ihn, Hong Koh, Keum Hwa Lee, Min Young Kim, Sinyoung Kim, Ji-Man Kang, Jun Yong Choi, Younhee Park, Myoung Soo Kim

**Affiliations:** Yonsei University College of Medicine, Seoul, Seoul-t'ukpyolsi, Republic of Korea; Severance Hospital, Yonsei University College of Medicine, Seoul, Seoul-t'ukpyolsi, Republic of Korea; Yonsei University College of Medicine, Seoul, Seoul-t'ukpyolsi, Republic of Korea; Severance Children’s Hospital, Yonsei University College of Medicine, Seoul, Seoul-t'ukpyolsi, Republic of Korea; Severance Children’s Hospital, Yonsei University College of Medicine, Seoul, Seoul-t'ukpyolsi, Republic of Korea; Severance Hospital, Yonsei University College of Medicine, Seoul, Seoul-t'ukpyolsi, Republic of Korea; Severance Children’s Hospital, Yonsei University College of Medicine, Seoul, Seoul-t'ukpyolsi, Republic of Korea; Yonsei University College of Medicine, Seoul, Seoul-t'ukpyolsi, Republic of Korea; Severance Hospital, Yonsei University College of Medicine, Seoul, Seoul-t'ukpyolsi, Republic of Korea; Severance Hospital, Yonsei University College of Medicine, Seoul, Seoul-t'ukpyolsi, Republic of Korea

## Abstract

**Background:**

Solid organ transplant (SOT) recipients are at high risk for severe COVID-19 due to immunosuppressant (IS) use. Compared to adult SOT recipients (SOTRs), studies on risk and vaccination effectiveness in pediatric SOTRs are limited. We aim to investigate the epidemiologic characteristics of COVID-19 and vaccine immunogenicity in pediatric SOTRs.

**Methods:**

This study was conducted at Severance Hospital in Korea, between 1999-2022. All SOTRs received SOT at the age ≤ 18 years were included. SOTRs who had a history of COVID-19 before SOT were excluded. Severe case was defined when there’s oxygen demand. Demographic data and information for risk factor exploration were retrospectively collected through chart review. Blood samples were collected prospectively after the COVID-19 vaccination policy for children was implemented. Humoral immunogenicity to WT, Delta and Omicron was investigated after 2 doses of BNT162b2 (BNT) using SARS-CoV-2 anti-S IgG titers, surrogate neutralization inhibition and plaque reduction neutralization tests. Post-vaccination samples of adult SOTRs were used as controls.

**Results:**

A total of 118 SOTRs were included. The median age at SOT was 5 years (1-11 years), and the male to female ratio was 1:1.1. About 77% (91/118) had COVID-19 and 11% (10/91) of them were hospitalized for COVID-19 management. Four cases were severe cases and there was no death. The number of IS was significantly associated with the number of infection (*p*=0.04), distinct from hospitalization and severity (*p*=0.82 and 0.50), and there was no significant difference in outcomes between SOTRs received ≥ 3 doses of the COVID-19 vaccine and those received 0-2 doses (*p*=1.00, 0.21 and 1.00, respectively). Multiple logistic regression showed age at SOT and re-SOT as factors associated with number of COVID-19 (*p*=0.03, CI 0.36-0.96 and *p*=0.03, CI 1.74-6431.8). In vaccine immunogenicity analysis, humoral responses in pediatric SOTR were not lower than in adult SOTRs (table 1). However, the vaccine immunogenicity against Omicron was very poor compared to WT or Delta (figure 1).

**Figure:**
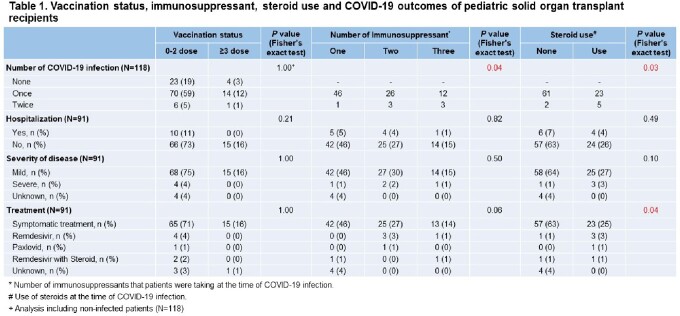

**Figure:**
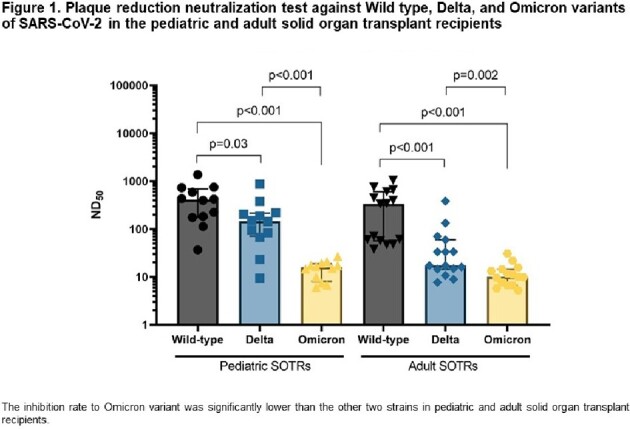

**Conclusion:**

Pediatric SOTR is susceptible to COVID, especially in older children at SOT and those who have undergone re-SOT. Two or Three doses of vaccination may be suboptimal for Omicron, so additional preventive measures are needed.

**Disclosures:**

**All Authors**: No reported disclosures

